# Eating disorders: the increase in requests for help and the optimization of resources

**DOI:** 10.1192/j.eurpsy.2024.1157

**Published:** 2024-08-27

**Authors:** M. Campana, M. Colombi, P. Milanese, A. Greco, C. Mucci

**Affiliations:** ^1^department of mental health and addictions, ASST Bergamo Est, Seriate; ^2^Department of Human and Social Sciences, University of Bergamo, Bergamo, Italy

## Abstract

**Introduction:**

This work aims to provide an updated overview of the eating disorders (EDs) which are a widespread pathology nowadays. Informations related to the clinical-nosographic characteristics, an in-depth analysis about systemic-relational theories and historical evolution are provided. In addition, current informations about epidemiological data, recovery, treatment related implications, new neuroscientific theories and risk factors are shown. Given the complexity of these disorders, the lack of resources and the the increasing demands for treatment, the main object is related to the construction of a questionnaire to manage the waiting lists.

**Objectives:**

Building a waiting list management model for EDs, Study and compare advantages and disadvantages of the source allocation ethical models (utilitarianism, prioritarianism, egalitarianism), Analyze EDs leading experts (doctors, dietitians, psychologists, psychiatrists) and EDs patients positioning with respect to priority treatment factors. Promote constructive dialog between EDs experts from different backgrounds and EDs patients.

**Methods:**

In order to know the various treatment alternatives available, the different levels and reference structures are illustrated. In addition, it is also suggested different reasoning based on the ethical models of egalitarianism, utilitarianism and prioritarianism in order to build a waiting list management model, which is the maximum goal of this work. This model needs to be supported by a series of validated tools such as the clinical interview and self-administered questionnaires to investigate psychopathological aspects and psychiatric symptoms. Going into more details, a questionnaire is proposed to the EDs leading experts, so that they can provide their own priority factors list and related thoughts in order to build “the most ethical” waiting list.

**Results:**

It is expected that both patients and clinicians tend to give priority to patients with greater psychophysical severity, not exclusively on the basis of physical parameters. Further hypothesis related to clinicians lead us believe that they tend to use utilitarian logics, in compliance with the demonstrated efficacy of early intervention. An evaluation that could lead to a disagreement between experts and patients is related to prioritize patients in the initial phase of the disease, which could be supported by clinicians, but not by patients, probably in connection with their personal experiences. In fact, this favoritism could have a negative impact on the care of the most serious cases who risk to be left to themselves.

**Conclusions:**

This work aims to encourage a constructive dialogue between experts and patients with EDs in order to build a functional intervention model which should be “the most ethical as possible” in order to save the greatest number of lives in respect of mental suffering.

**Disclosure of Interest:**

None Declared

